# Recent advances in genetic etiology of non-syndromic deafness in children

**DOI:** 10.3389/fnins.2023.1282663

**Published:** 2023-10-19

**Authors:** Yawei Feng, Sunyi Hu, Shen Zhao, Ming Chen

**Affiliations:** Xiangyang Central Hospital, Affiliated Hospital of Hubei University of Arts and Science, Xiangyang, China

**Keywords:** genetic hearing loss, deafness genes, non-syndromic, genetic etiology, children

## Abstract

Congenital auditory impairment is a prevalent anomaly observed in approximately 2–3 per 1,000 infants. The consequences associated with hearing loss among children encompass the decline of verbal communication, linguistic skills, educational progress, social integration, cognitive aptitude, and overall well-being. Approaches to reversing or preventing genetic hearing loss are limited. Patients with mild and moderate hearing loss can only use hearing aids, while those with severe hearing loss can only acquire speech and language through cochlear implants. Both environmental and genetic factors contribute to the occurrence of congenital hearing loss, and advancements in our understanding of the pathophysiology and molecular mechanisms underlying hearing loss, coupled with recent progress in genetic testing techniques, will facilitate the development of innovative approaches for treatment and screening. In this paper, the latest research progress in genetic etiology of non-syndromic deafness in children with the highest incidence is summarized in order to provide help for personalized diagnosis and treatment of deafness in children.

## Introduction

Congenital deafness is a common birth defect. It is estimated that there are 2–3 clinically deaf babies for every 1,000 births (Gaffney et al., 2010; Korver et al., 2017). Congenital deafness can be categorized into different levels of severity, namely mild (26–40 decibels), moderate (41–55 decibels), moderately severe (56–71 decibels), profound (71–90 decibels), and severe (greater than 91 decibels). In the case of children, approximately 29% experience unilateral hearing loss while bilateral hearing loss is present in about 71% (van Beeck Calkoen et al., 2019). The incidence rate of congenital permanent bilateral hearing loss is even lower, affecting only 1.2 per 1,000 births (Morton and Nance, 2006). Congenital deafness has multifactorial etiology and regrettably lacks a definitive cure. However, cochlear implants and hearing aids serve as the primary modalities employed to ameliorate the quality of life for children with auditory impairment, but these do not fundamentally restore hearing. Even with a cochlear implant, it produces hearing that mimics only a small part of the sound that can be perceived by a healthy ear. Congenital deafness contributes in part to children’s speech impairments and social and cognitive impairments (Lieu et al., 2020). Early prevention and diagnosis of congenital hearing loss is critical, making universal newborn hearing screening of great value (Morton and Nance, 2006; Katbamna et al., 2008). In addition to conventional audiometry in screening, molecular genetic screening is evolving (Smith, 2004), making it particularly important to explore the molecular pathogenesis of congenital hearing loss.

Hearing loss can be caused by a variety of environmental and genetic factors, statistically equal parts of each (Gaffney et al., 2010). Among the environmental factors are infections, drugs or trauma. Vertical mother-to-child transmission is a common neonatal infection factor. Infectious viruses commonly transmitted to the fetus through teratogenic effects include TORCH (Toxoplasmosis-Other-Rubella-Cytomegalovirus-Herpes Simplex) (Nance, 2003). Among these, congenital hearing loss can be attributed to cytomegalovirus, rubella, herpes simplex, lymphocytic choroidal meningitis, and Zika virus infections (Macedo-da-Silva et al., 2020). Other environmental factors occurring at or after birth that can lead to hearing loss include hypoxia, prematurity, complications related to Rh factors in the blood, and bacterial meningitis. They usually result in a degree of sensorineural hearing loss that ranges from mild to severe. The hearing process involves at least 1% of human genes (about 300 genes), and alterations in any single gene or regulatory element can lead to hearing loss (Friedman and Griffith, 2003). The hereditary causes of congenital deafness are mainly categorized into syndromes and non-syndromes. The former accounts for 30% of cases with hereditary causes and is accompanied by other symptoms in addition to deafness, while the latter accounts for 70% of cases where deafness is the only symptom (Smith et al., 2005; Lammens et al., 2013).

The majority of cases involving hereditary auditory impairment are not accompanied by additional symptoms. Approximately 80% of non-syndromic cases can be attributed to autosomal recessive hearing loss (ARNSHL), while autosomal dominant hearing loss (ADNSHL) accounts for 20%, and X-linked or mitochondrial inheritance contributes to approximately 1–2% of the total number of cases (Hilgert et al., 2009). To date, 124 genes have been reported to be associated with nonsyndromic hearing loss.^1^ ARNSHL is highly genetically heterogeneous, with approximately 89 genetic loci and 76 mutations associated with the etiology of ARNSHL (Imtiaz, 2022). The primary etiology of ARNSHL cases is attributed to genetic mutations in GJB2, while the gene SLC26A4 ranks as the second most prevalent factor associated with ARNSHL. Other ARNSHL-related genes include MYO15A, OTOF, CDH23 and TMC1 (Hilgert et al., 2009). Currently, approximately 70 loci and 51 genes are associated with ADNSHL, mainly including TECTA, WFS1, KCNQ4, COCH and GJB2 (Hildebrand et al., 2011). Five genes and six loci have been identified as being associated with X-linked nonsyndromic hearing loss, including PRPS1, POU3F4, SMPX, AIFM1and COL4A6(de Kok et al., 1995; Liu et al., 2010; Huebner et al., 2011; Schraders et al., 2011; Zong et al., 2015).

However, the primary pathologic mechanisms by which these genes contribute to hearing loss are still unclear. Therefore, we review the genes associated with nonsyndromic hearing loss in recent years. We explore the causes of congenital deafness and clarify their pathologic mechanisms, aiming to provide assistance in the clinical diagnosis and treatment of congenital deafness.

## Genetic causes of deafness

Over 180 genes associated with deafness have been identified and investigated in past studies (Ma et al., 2023). Hereditary deafness can be categorized into four main groups based on their pathomechanisms: ciliopathy, neuropathy, synaptopathy, neuropathies, or homeostasis disorders.

Cochlear ciliopathy refers to a condition resulting from the disruption of normal ciliary function. These disorders, known as cochlear ciliopathies, affect the growth and maintenance of bundles of mechanosensory stereocilia on the apical surface of sensory hair cells that are crucial for auditory perception. Although stereocilia themselves are not classified as cilia, their proper development and alignment depend on primary cilia present in hair cells (Pollock and McDermott Jr., 2015); Auditory neuropathy is a condition characterized by impaired transmission of auditory signals from the cochlea to the brain, resulting from disrupted nerve impulses between the spiral ganglion and the central auditory pathway. Consequently, this disruption can lead to hearing loss. It is worth noting that disturbances in inner ear equilibrium may also contribute to auditory impairments. Cellular connections, ion channels, and their regulators are essential for the development of endolymphatic structures critical to auditory function. Moreover, neuropathy is characterized by aberrations in neural crest cell differentiation, which significantly impact the formation of various normal ear components including cartilage, auditory ossicles, cochlear glial cells, and interneurons involved in hearing (Ritter and Martin, 2019). All of these disorders may be recessively or dominantly inherited and result in hearing loss.

Individuals with nonsyndromic deafness have only symptoms of deafness. In most cases, the onset of non-syndromic deafness in patients with autosomal dominant inheritance occurs in the first to fourth decade of life (Van Laer et al., 1999). Autosomal recessive nonsyndromic deafness, on the other hand, is congenital or prelingual, and most of the time it leads to severe hearing loss (Sundstrom et al., 1999). Nonsyndromic deafness disorders can be classified by their locus names, with autosomal dominant abbreviations DFNA and autosomal recessive abbreviations DFNB, followed by a number representing the order in which they are found. Some DFNAs are located in the same spot on the chromosome as DNFBs because certain genes may carry both recessive and dominant variants. To date, there have been more than 180 loci associated with hearing loss reported, but only 121 genes associated with deafness are known. Several common genes associated with nonsyndromic deafness will be presented below (Table 1).

**Table 1:** Several common genes associated with nonsyndromic hearing loss.

Function	Gene	Location	Pubmed
Electrolyte recycling	*GJB2*	13q11-q12	8136828;37373495;37333892
	*GJB3*	1p35.1	9843210;37373495
	*GJB6*	13q12	10471490;37373495
	*Connexin 26*		9139825;37373495
Changes in ATP release and Ca + signaling	*SLC26A4*	7q31	8541853;36362242
	*KCNQ1*		36140355
	*KCNA4*	1p34	8035838;36140355
Auditory neuropathy	*OTOF*	2p22-p23	8789454;37189200
	*DFNB59*	2q31.1-q31.3	16804542
Cytoskeletal alterations of inner ear hair cells	*MYO7A*	11q12.3-q21	9171832;34979615
	*MYO15A*	17p11.2	7704031;37189200
	*CLDN14*	21q22	31781163
	*DIAPH1*	5q31	1350680;35060117

## *GJB2*, *GJB3*, and *GJB6*

The connexin gene family is the most common gene contributing to hearing loss, with mutations in Connexin 26 being the major protein responsible for non-characteristic hearing loss and the most abundantly expressed connexin in the inner ear (Cohn and Kelley, 1999; Chai et al., 2022; Zong et al., 2023). Connexin 26 can bind to itself or to other connexins to form channels that connect exons. Two homologous or heterologous connexins on neighboring cells can form gap junctions through which ions and small molecules can pass for intercellular communication. The presence of connexin 26 proteins was demonstrated in Sertoli cells, as well as in sulcus and border cells within the organ of Corti, and also in the lateral wall of the cochlea, based on a rat study. Therefore, it can be inferred that both the epithelial gap junction system and connective tissue gap junction system exist within the cochlea (Kikuchi et al., 1995).

**Figure 1:**
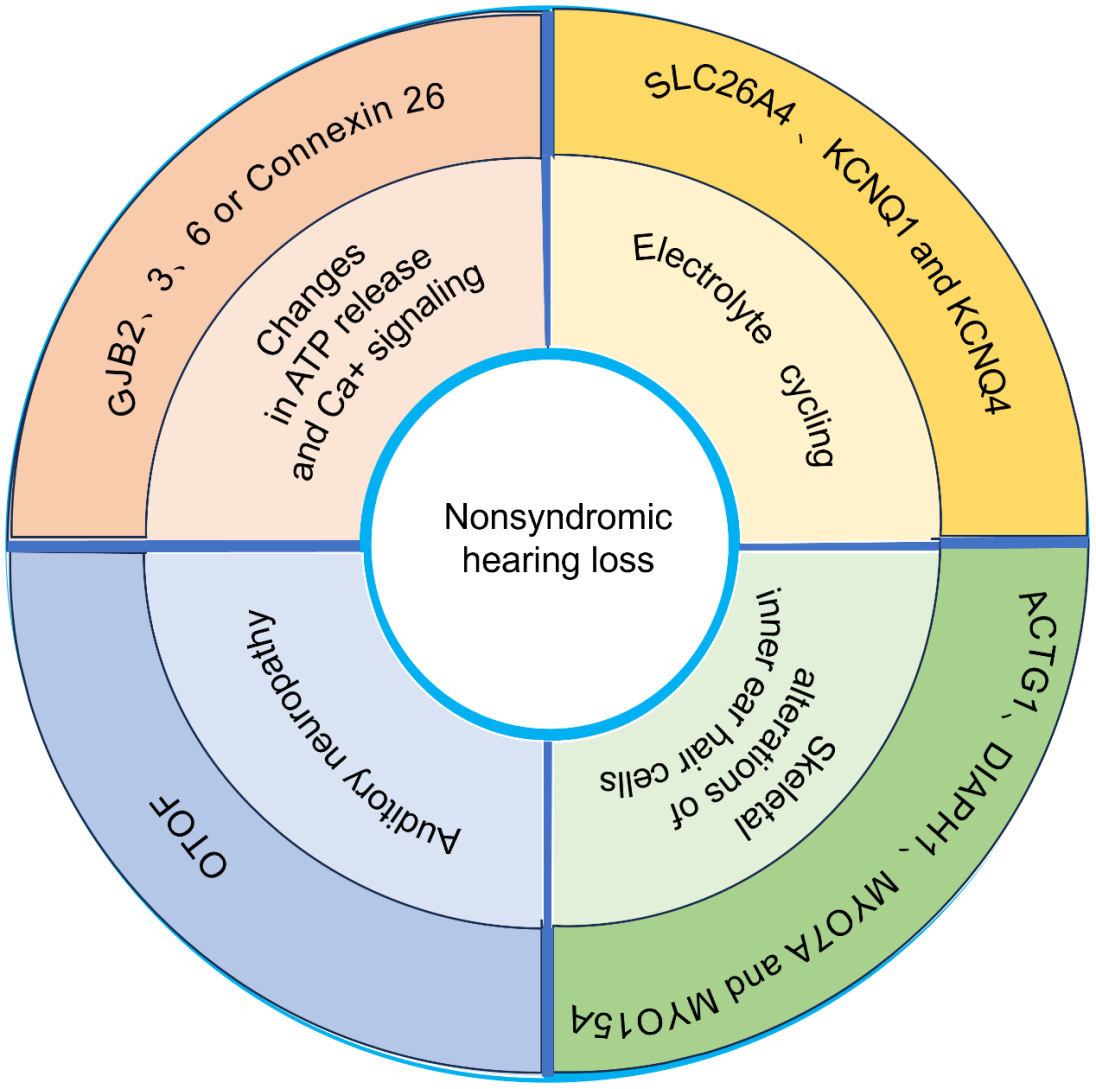
Schematic representation of several common genes associated with nonsyndromic hearing loss.

*GJB2* encodes connexin 26 protein (Kiang et al., 1997). *GJB2* gene mutations can cause hearing loss, both recessive and dominant, mainly due to mutations in the *DFNB1A* or *DFNA3* motifs (Kelsell et al., 1997). Mutations in the *GJB2* gene lead to 50% of autosomal recessive non-syndromic hearing disorders in Europe (Al Mutery et al., 2022). Global research findings estimate the occurrence rate of autosomal recessive hearing loss caused by *GJB2* mutations to be approximately 16.9% (Chan and Chang, 2014), with Europe exhibiting the highest prevalence (27.1%) and sub-Saharan Africa displaying the lowest prevalence (5.6%). Distinct causative mutations have been extensively investigated in various populations, including 35delG in Europe, 235delC in East Asia, and W24X in India. These documented mutations could potentially be regarded as founding variants (Buonfiglio et al., 2020). One recent study showed the presence of *GJB2* or *GJB6* mutations in 38% of patients with non-syndromic hearing loss in Argentina (Rabionet et al., 2000). Many genetic alterations in *GJB2* causing deafness have been described so far (Figure 1).

In an animal laboratory study, it was observed that mice conditioned to interfere with *GJB2* exhibited significant hearing loss and displayed signs of hair cell apoptosis and degeneration in Sertoli cells. The process of apoptosis initiated shortly after the onset of auditory function, suggesting that exposure to sound stimulation may trigger this apoptotic response. A significant decrease in cochlear potential and endolymphatic potassium concentration was observed within the cochlea. It is hypothesized that the absence of connexin 26 disrupts the movement of K+ ions, impeding glutamate absorption due to elevated extracellular K+ levels, ultimately leading to apoptosis of hair cells (Teubner et al., 2003). Connexin 26-based gap junction channels facilitate the transfer of small molecules, such as glucose, ions, second messengers, and other substances. Consequently, *GJB2*-deficient mice exhibit prenatal mortality during the early to mid-pregnancy stage due to significantly compromised nutrient absorption capacity for essential substances like glucose and others, ultimately resulting in fetal demise (Gabriel et al., 1998). Deletion of Connexin 26, which is abundant in inner ear cells, also leads to impaired nutrient delivery to inner ear cells. All of these contribute to the development of hearing loss (Wang et al., 2023).

Connexin 31 and connexin 30 are encoded by the *GJB3* and *GJB6* genes, respectively. These genes have been implicated in hereditary hearing loss. The etiology of autosomal dominant deafness, known as *DFNA2B*, associated with mutations in the *GJB3* gene remains unknown. Conversely, mutations in the *GJB6* gene can result in both autosomal recessive deafness (*DFNB1B*) and autosomal dominant deafness (*DFNA3B*) (Soleimani et al., 2001). Mice lacking *GJB6* exhibit significant structural hearing damage, while their cochlear and vestibular end-organs develop normally. These mice experience a lack of cochlear potential from the onset of hearing, which is crucial for high sensitivity in mammalian auditory systems. After 18 generations, initiation of apoptosis was observed in sensory epithelial cells within the cochlea, suggesting that *GJB6* plays a critical role in generating intracochlear potentials and maintaining survival of auditory hair cells once hearing has commenced (Bidart et al., 2000).

However, the underlying mechanism of how mutations in the *GJB* gene lead to deafness remains unclear, and several current findings contradict previous hypotheses, which remain to be clarified by further studies.

## *SLC26A4*, *KCNQ1*, and *KCNQ4*

The gene *SLC26A4* is responsible for encoding pendrin, a transmembrane transporter that facilitates the transport of anions (such as chloride ions, iodide ions, and bicarbonate ions) across cellular membranes (Soleimani et al., 2001). This gene exhibits elevated expression levels in specific organs including the cochlea, thyroid gland, and renal tubules, Its mutations can lead to autosomal recessive inheritance (Bidart et al., 2000). Pituitary adrenocorticotropic hormone dysfunction leads to impaired hearing in patients with Pender syndrome (PDS) and non-syndrome associated with dilated vestibular aqueduct (*EVA*) (*DFNB4*) (Campbell et al., 2001). *SLC26A4*-associated hearing loss is associated with inner ear malformations, hearing loss, vestibular dysfunction, and thyroid abnormalities (Honda and Griffith, 2022). *SLC26A4* deficient mice exhibited profound hearing loss and impairment in their vestibular system (Everett et al., 2001). The underlying molecular mechanism responsible for the enlargement of endolymphatic fluid may be attributed to the compromised ability to absorb ions and water from the inner cavity during the development of endolymphatic sacs (Choi et al., 2011). The study revealed that the *SLC26A4* knockout mouse model exhibited a more pronounced auditory and inner ear phenotype compared to individuals with hearing loss associated with *SLC26A4* (Lu et al., 2011; Wen et al., 2019).

*KCNQ*, a cluster of potassium channels, is associated with various medical conditions such as auditory impairment and cardiac arrhythmia (Robbins, 2001). The *KCNQ* protein is a membrane potential-dependent voltage-gated channel that can be activated upon cell membrane depolarization. The normal functioning of auditory perception relies on the presence of *KCNQ1* and *KCNQ4*, two pivotal members within the *KCNQ* family, they can cause autosomal dominant deafness. This underscores the critical role of potassium in maintaining fluid homeostasis and its dynamic nature within the inner ear (Reardon et al., 1993; Nie, 2008). Kv7.1 and Kv7.4 are potassium channels that undergo voltage-gating, which are encoded by *KCNQ1* and *KCNQ4* genes, respectively. While *KCNQ1* has been associated with both cardiovascular diseases and hearing loss, *KCNQ4* is specifically linked to auditory impairment (Maljevic et al., 2010). Interference with KCNQ1 gene expression in mice results in deafness and severe inner ear morphologic abnormalities (Rivas and Francis, 2005). *KCNQ4* is expressed in sensory outer hair cells, and mutations in its gene lead to *DFNA2* (Kharkovets et al., 2000). It allows potassium to flow out of the extracellular space to restore the cell to an excited state. In mice, disruption of *KCNQ4* channels leads to damage in the outer hair cells. The pathomechanism of hearing loss due to *KCNQ* is usually explained by dominant-negative inhibition or haploinsufficiency (Wang and Li, 2016; Homma, 2022).

## *OTOF* and *DFNB59*

Another pivotal factor contributing to recessive hearing loss is the presence of mutations in the *OTOF* gene, commonly referred to as *DFNB9*. Multiple studies conducted across diverse regions have indicated that alterations in the pathogenic *OTOF* gene account for approximately 2.3 to 7.3% of cases involving recessive hearing impairment (Duman et al., 2011; Iwasa et al., 2019). The *OTOF* gene comprises 28 exons that encode diverse long and short isotypes (Yasunaga et al., 1999). Studies in affected families have shown that long isotypes are necessary for hearing function (Yasunaga et al., 2000). Pathogenic mutations in genes display a wide distribution, with the majority being unique to individual families. Furthermore, there have been instances of recurring variants identified across diverse racial populations. *Q829X* was found in the Spanish population and *p.R1939Q* in the Japanese population among the most common mutations (Iwasa et al., 2013). The *OTOF* gene encodes otoferlin, which is a key protein for synapses in auditory sensory cells (Yasunaga et al., 1999). Deficiency of otoferlin leads to impaired release of synaptic vesicles at synapses in inner hair cells, which manifests as auditory synucleinopathy (Pangrsic et al., 2010).

Cochlear cell abnormalities are the most common cause of hereditary sensorineural hearing loss. However, lesions other than those of the cochlea account for a sizable part of cases, up to 10% of which result in permanent hearing loss in youngsters. Otoacoustic emission (OAE), which is low-level sound produced in the cochlea as a result of mechanical activity of OHCs, and recording auditory brainstem response (ABR), which measures the electrophysiological response evoked by acoustic stimulation of the auditory nerve and brainstem, are clinical tests for sensorineural hearing impairment. With auditory neuropathy, the outer hair cells of the cochlea are still functional but the ABR is absent or badly distorted while the OAE is preserved. Auditory neuropathy affects the neurotransmission of auditory signals. The first gene associated with cochlear cell disease in humans is called DFNB59, and it is found on chromosome 2q31.1–q31.3. This gene was first identified as the source of four congenic families’ autosomal recessive hereditary auditory neuropathy, and it has since been linked to autosomal recessive inheritance in numerous other families (Delmaghani et al., 2006). Pejvakin, a DFNB59 product, plays a significant function in the physiology of auditory neurons and is expressed in all relays of the afferent auditory route from the cochlea to the midbrain (Delmaghani et al., 2006).

Pejvakin, a 352-residue protein, has been linked to the oxidative stress-induced proliferation of this organelle, according to studies. Peroxisomes play a vital role in homeostasis and defense against noise-induced hearing loss in the auditory system, as shown by pejvakin defective mice (Delmaghani et al., 2015). It is thought that the following factors play a major part in hearing loss. (1) Pejvakin deficiency results in peroxisome anomalies in cochlear hydroxy carbon after hearing onset, impairing the cochlear antioxidant defense and causing damage to auditory hair cells as a result of reactive oxygen species (ROS) (Delmaghani et al., 2015; Defourny et al., 2019); (2) A significant amount of phenotypic heterogeneity in hearing is caused by pejvakin loss in mice (Delmaghani et al., 2015); (3) The Pejvakin deficiency makes one more susceptible to low energy (Delmaghani et al., 2015); (4) Loss of pejvakin impairs the propagation of action potentials in the auditory pathway following controlled electrical and acoustic exposures, as shown by decreased E II wave amplitude and increased E II-E IV and ABRI-IV wave intervals in PIVK-mice. (5) Loss of pejvakin renders auditory pathway neurons extremely susceptible to exposure to mild, brief stimuli (Delmaghani et al., 2015). In conclusion, DFNB59 mutations harm sensory hair cells in addition to causing neurological abnormalities.

## *ACTG1*, *DIAPH1*, *MYO7A*, *MYO15A*, *CLDN14*, and *GRHL2*

The cytoskeleton is a complex network of interconnected filaments and tubules that extend from the nucleus to the plasma membrane (Hoyt et al., 1997), consisting of intermediate filaments, microtubules, and actin filaments. Numerous genes associated with hereditary hearing loss have been linked to the cytoskeleton, including *ACTG1* (*DFNA20/26*), which encodes gamma actin; *DIAPH1* (*DFNA1*), which regulates actin filament polymerization; *ESPN* (*DFNB36*), involved in producing actin bundles; *RDX (DFNB24)*, facilitating connections between actin filaments and stereocilia. Additionally, several unconventional myosin-encoding genes such as *MYO7A* (*DFNA11, DFNB2, Usher syndrome 1B*)*, MYO6* (*DFNA22* and *DFNB37*) and *MYO15A* (*DFNB3*) are also implicated.

The gene *ACTG1* is responsible for the production of gamma actin, predominantly found in the hair cells of the inner ear. Mutations in this gene have been associated with autosomal dominant inherited hearing loss (known as *DFNA20/DFNA26*) (van Wijk et al., 2003). Exposure to noise or aging can cause damage to the cochlear structure and disrupt various processes such as bundling, gelation, polymerization, or myosin movement within hair cells (Morin et al., 2009). Consequently, this impedes their self-repair capacity and leads to a gradual decline in auditory function over time. Recent findings from a DNA sequencing study suggest that defective gamma actin may impair proper formation of F-actin, thereby contributing to the pathogenesis of *ACTG1* mutations (Miyajima et al., 2020).

*DIAPH1* plays a crucial role in the regulation of actin polymerization within the hair cells of the inner ear. The expression of DIAPH1 is predominantly observed in the inner pillar cells, as well as the basal and outer pillar cells of outer hair cells (Neuhaus et al., 2017). Mutations in *DIAPH1* are responsible for autosomal dominant hearing loss (*DFNA1*) (Lynch et al., 1997).

The unconventional myosin proteins encoded by *MYO7A* and *MYO15A* are implicated in Usher syndrome, accounting for approximately 50% of cases (Millan et al., 2011). Furthermore, mutations in the *MYO7A* gene can result in autosomal recessive nonsyndromic hearing impairment. Animal models such as Shaker-1 and headband mice carrying the *MYO7A* mutation have exhibited symptoms indicative of vestibular dysfunction, including hyperactivity, head shaking, and head twisting. These models also demonstrate progressive degeneration of the organ of Corti (Gibson et al., 1995). In hair mice with cephalic abnormalities, outer hair cells exhibit O-shaped stereocilia instead of V-shaped ones, while inner hair cells present giant stereocilia (Rhodes et al., 2004). These findings suggest that defects in stereocilia morphogenesis contribute to both vestibular dysfunction and deafness.

Autosomal recessive hearing impairment, known as *DFNB3*, is caused by mutations in the *MYO15A* gene. *MYO15A* plays a crucial role in the elongation and development of stereocilia and actin filaments. The interaction between retention factors and *MYO15A* is essential for maintaining the cohesion of stereocilia structures (Belyantseva et al., 2005). Mutations in the *MYO7A* gene were initially identified in Indonesian families, resulting in shortened stereocilia associated with cochlear and vestibular dysfunction (Anderson et al., 2000). These findings suggest that alterations caused by *MY015* may disrupt both the structural integrity and functional capabilities within the sensory epithelium.

Mutations in the *CLDN14* gene are responsible for *DFNB29*, an autosomal recessive nonsyndromic hearing loss disorder (Ben-Yosef et al., 2003). The expression of *CLDN14* is primarily observed in the cochlea, liver, and kidney. This gene encodes claudin-14, a tight junction protein that enhances trans-epithelial resistance by reducing cation permeability, particularly potassium ions. Insufficient production of claudin 14 leads to inner hair cell deterioration and rapid outer hair cell death (Lee et al., 2012).

*GRHL2* exhibits widespread expression across various human tissues, including the prostate, thymus, kidney, lung, salivary gland, mammary gland, and digestive tract. Mutations in *GRHL2* have been associated with *DFNA 28* (Autosomal dominant nonsyndromic tone neuropathy hearing loss) (Peters et al., 2002; Petrof et al., 2014), resulting in notable phenotypic changes such as enlarged ear sacs, smaller or absent otoliths, malformed semicircular canals, insensitivity to acoustic stimuli, and impaired swimming maneuvers. In embryonic ear epithelial cells affected by *GRHL2* mutations, there is a significant reduction or elimination of claudin-b and EpCAM expression while exhibiting aberrant formation of the apical junction complex (Han et al., 2011).

## Summary and outlook

In summary, the main pathological mechanisms of hearing loss may focus on the following: (1) Electrolyte cycling, including imbalance and impaired uptake of K^+^, Na^+^, and Cl^+^, etc.(2) Changes in ATP release and Ca^+^ signaling, which may lead to impaired development of the columnar cytoskeleton as well as cochlea development (Bobbin and Thompson, 1978; Kujawa et al., 1994; Munoz et al., 1995) (3) Auditory neuropathy (mainly caused by mutations in the OTOF gene). (4) Cytoskeletal alterations of inner ear hair cells. Different genetic alterations lead to different pathologic phenotypes. The occurrence of hearing loss due to GJB2 mutations has been found to precede hair cell degeneration, as indicated by recent studies. This finding suggests that K + -cycling is unlikely to be the underlying mechanism responsible for GJB2-related hearing impairment (Liang et al., 2012).

Congenital hearing loss is the most prevalent congenital defect, affecting two to three out of every 1,000 newborns. The etiology of congenital deafness is multifactorial and can be attributed to both genetic and environmental factors. In recent decades, genetic studies on hearing loss have yielded valuable insights into the molecular basis, development, and function of the auditory system. Furthermore, recent technological advancements have significantly improved our ability to accurately diagnose various forms of hereditary deafness at a molecular level. At present, we can use Sanger sequencing, Gene chip and other hot spot mutation screening technology, Deafness gene targeted capture sequencing (Panel sequencing), Whole exome sequencing, Whole genome sequencing, Whole genome scanning, and other genetic testing methods (such as Multiplex Ligation-dependent Probe Amplification, MLPA) and other technologies to improve our early identification and diagnosis of hereditary deafness.

However, despite the numerous advancements made in this field, certain limitations exist. The timely detection and intervention of congenital hearing impairment in newborns can significantly enhance their linguistic and verbal development while also improving their motor, cognitive, and social capabilities. Therefore, it is imperative to prioritize active screening for hearing loss in infants as well as molecular genetic screening procedures. Identifying the etiology of congenital deafness in some cases will help to achieve the best therapeutic effect and personalize each etiology in the future.

## Author contributions

YF: Data curation, Formal analysis, Investigation, Methodology, Writing – original draft. SH: Data curation, Formal analysis, Methodology, Software, Writing – original draft. SZ: Conceptualization, Project administration, Supervision, Validation, Visualization, Writing – review & editing. MC: Conceptualization, Funding acquisition, Project administration, Supervision, Validation, Visualization, Writing – review & editing.

## Footnotes

^1^https://hereditaryhearingloss.org/

## References

[ref1] Al Mutery A. Mahfood M. Chouchen J. Tlili A. (2022). Genetic etiology of hereditary hearing loss in the Gulf cooperation council countries. Hum. Genet. 141, 595–605. doi: 10.1007/s00439-021-02323-x, PMID: 34338889

[ref2] Anderson D. W. Probst F. J. Belyantseva I. A. Fridell R. A. Beyer L. Martin D. M.. (2000). The motor and tail regions of myosin XV are critical for normal structure and function of auditory and vestibular hair cells. Hum. Mol. Genet. 9, 1729–1738. doi: 10.1093/hmg/9.12.1729, PMID: 10915760

[ref3] Belyantseva I. A. Boger E. T. Naz S. Frolenkov G. I. Sellers J. R. Ahmed Z. M.. (2005). Myosin-XVa is required for tip localization of whirlin and differential elongation of hair-cell stereocilia. Nat. Cell Biol. 7, 148–156. doi: 10.1038/ncb1219, PMID: 15654330

[ref4] Ben-Yosef T. Belyantseva I. A. Saunders T. L. Hughes E. D. Kawamoto K. Van Itallie C. M.. (2003). Claudin 14 knockout mice, a model for autosomal recessive deafness DFNB29, are deaf due to cochlear hair cell degeneration. Hum. Mol. Genet. 12, 2049–2061. doi: 10.1093/hmg/ddg210, PMID: 12913076

[ref5] Bidart J. M. Mian C. Lazar V. Russo D. Filetti S. Caillou B.. (2000). Expression of pendrin and the Pendred syndrome (PDS) gene in human thyroid tissues. J. Clin. Endocrinol. Metab. 85, 2028–2033. PMID: 10843192 10.1210/jcem.85.5.6519

[ref6] Bobbin R. P. Thompson M. H. (1978). Effects of putative transmitters on afferent cochlear transmission. Ann. Otol. Rhinol. Laryngol. 87, 185–190. PMID: 206175 10.1177/000348947808700207

[ref7] Buonfiglio P. Bruque C. D. Luce L. Giliberto F. Lotersztein V. Menazzi S.. (2020). GJB2 and GJB6 genetic variant curation in an Argentinean non-syndromic hearing-impaired cohort. Genes 11:1233. doi: 10.3390/genes11101233, PMID: 33096615 PMC7589744

[ref8] Campbell C. Cucci R. A. Prasad S. Green G. E. Edeal J. B. Galer C. E.. (2001). Pendred syndrome, DFNB4, and PDS/SLC26A4 identification of eight novel mutations and possible genotype-phenotype correlations. Hum. Mutat. 17, 403–411. doi: 10.1002/humu.1116, PMID: 11317356

[ref9] Chai R. Li H. Yang T. Sun S. Yuan Y. (2022). Editorial: hearing loss: mechanisms and prevention. Front. Cell Dev. Biol. 10:838271. doi: 10.3389/fcell.2022.838271 35186939 PMC8850831

[ref10] Chan D. K. Chang K. W. (2014). GJB2-associated hearing loss: systematic review of worldwide prevalence, genotype, and auditory phenotype. Laryngoscope 124, E34–E53. doi: 10.1002/lary.24332, PMID: 23900770

[ref11] Choi B. Y. Kim H. M. Ito T. Lee K. Y. Li X. Monahan K.. (2011). Mouse model of enlarged vestibular aqueducts defines temporal requirement of Slc26a4 expression for hearing acquisition. J. Clin. Invest. 121, 4516–4525. doi: 10.1172/JCI59353, PMID: 21965328 PMC3204851

[ref12] Cohn E. S. Kelley P. M. (1999). Clinical phenotype and mutations in connexin 26 (DFNB1/GJB2), the most common cause of childhood hearing loss. Am. J. Med. Genet. 89, 130–136. doi: 10.1002/(SICI)1096-8628(19990924)89:3<130::AID-AJMG3>3.0.CO;2-M 10704187

[ref13] de Kok Y. J. van der Maarel S. M. Bitner-Glindzicz M. Huber I. Monaco A. P. Malcolm S.. (1995). Association between X-linked mixed deafness and mutations in the POU domain gene POU3F4. Science 267, 685–688. doi: 10.1126/science.7839145, PMID: 7839145

[ref14] Defourny J. Aghaie A. Perfettini I. Avan P. Delmaghani S. Petit C. (2019). Pejvakin-mediated pexophagy protects auditory hair cells against noise-induced damage. Proc. Natl. Acad. Sci. U. S. A. 116, 8010–8017. doi: 10.1073/pnas.1821844116, PMID: 30936319 PMC6475433

[ref15] Delmaghani S. Defourny J. Aghaie A. Beurg M. Dulon D. Thelen N.. (2015). Hypervulnerability to sound exposure through impaired adaptive proliferation of peroxisomes. Cells 163, 894–906. doi: 10.1016/j.cell.2015.10.023, PMID: 26544938

[ref16] Delmaghani S. del Castillo F. J. Michel V. Leibovici M. Aghaie A. Ron U.. (2006). Mutations in the gene encoding pejvakin, a newly identified protein of the afferent auditory pathway, cause DFNB59 auditory neuropathy. Nat. Genet. 38, 770–778. doi: 10.1038/ng1829, PMID: 16804542

[ref17] Duman D. Sirmaci A. Cengiz F. B. Ozdag H. Tekin M. (2011). Screening of 38 genes identifies mutations in 62% of families with nonsyndromic deafness in Turkey. Genet. Test. Mol. Biomarkers 15, 29–33. doi: 10.1089/gtmb.2010.0120, PMID: 21117948

[ref18] Everett L. A. Belyantseva I. A. Noben-Trauth K. Cantos R. Chen A. Thakkar S. I.. (2001). Targeted disruption of mouse Pds provides insight about the inner-ear defects encountered in Pendred syndrome. Hum. Mol. Genet. 10, 153–161. doi: 10.1093/hmg/10.2.153 11152663

[ref19] Friedman T. B. Griffith A. J. (2003). Human nonsyndromic sensorineural deafness. Annu. Rev. Genomics Hum. Genet. 4, 341–402. doi: 10.1146/annurev.genom.4.070802.110347 14527306

[ref20] Gabriel H. D. Jung D. Butzler C. Temme A. Traub O. Winterhager E.. (1998). Transplacental uptake of glucose is decreased in embryonic lethal connexin26-deficient mice. J. Cell Biol. 140, 1453–1461. doi: 10.1083/jcb.140.6.1453, PMID: 9508777 PMC2132681

[ref21] Gaffney M. Eichwald J. Grosse S. D. Mason C. A. Centers for Disease Control and Prevention (2010). Identifying infants with hearing loss - United States, 1999–2007. MMWR Morb. Mortal. Wkly. Rep. 59, 220–223. 20203554

[ref22] Gibson F. Walsh J. Mburu P. Varela A. Brown K. A. Antonio M.. (1995). A type VII myosin encoded by the mouse deafness gene shaker-1. Nature 374, 62–64. doi: 10.1038/374062a0, PMID: 7870172

[ref23] Han Y. Mu Y. Li X. Xu P. Tong J. Liu Z.. (2011). Grhl2 deficiency impairs otic development and hearing ability in a zebrafish model of the progressive dominant hearing loss DFNA28. Hum. Mol. Genet. 20, 3213–3226. doi: 10.1093/hmg/ddr234, PMID: 21610158

[ref24] Hildebrand M. S. Morin M. Meyer N. C. Mayo F. Modamio-Hoybjor S. Mencia A.. (2011). DFNA8/12 caused by TECTA mutations is the most identified subtype of nonsyndromic autosomal dominant hearing loss. Hum. Mutat. 32, 825–834. doi: 10.1002/humu.21512, PMID: 21520338 PMC3326665

[ref25] Hilgert N. Smith R. J. Van Camp G. (2009). Function and expression pattern of nonsyndromic deafness genes. Curr. Mol. Med. 9, 546–564. doi: 10.2174/156652409788488775, PMID: 19601806 PMC2840995

[ref26] Hilgert N. Smith R. J. H. Van Camp G. (2009). Forty-six genes causing nonsyndromic hearing impairment: which ones should be analyzed in DNA diagnostics? Mutat. Res. 681, 189–196. doi: 10.1016/j.mrrev.2008.08.002, PMID: 18804553 PMC2847850

[ref27] Homma K. (2022). The pathological mechanisms of hearing loss caused by KCNQ1 and KCNQ4 variants. Biomedicine 10:2254. doi: 10.3390/biomedicines10092254, PMID: 36140355 PMC9496569

[ref28] Honda K. Griffith A. J. (2022). Genetic architecture and phenotypic landscape of SLC26A4-related hearing loss. Hum. Genet. 141, 455–464. doi: 10.1007/s00439-021-02311-1, PMID: 34345941

[ref29] Hoyt M. A. Hyman A. A. Bahler M. (1997). Motor proteins of the eukaryotic cytoskeleton. Proc. Natl. Acad. Sci. U. S. A. 94, 12747–12748. doi: 10.1073/pnas.94.24.12747, PMID: 9398068 PMC34170

[ref30] Huebner A. K. Gandia M. Frommolt P. Maak A. Wicklein E. M. Thiele H.. (2011). Nonsense mutations in SMPX, encoding a protein responsive to physical force, result in X-chromosomal hearing loss. Am. J. Hum. Genet. 88, 621–627. doi: 10.1016/j.ajhg.2011.04.007 21549336 PMC3146719

[ref31] Imtiaz A. (2022). ARNSHL gene identification: past, present and future. Mol. Gen. Genomics 297, 1185–1193. doi: 10.1007/s00438-022-01926-x 35869994

[ref32] Iwasa Y. I. Nishio S. Y. Sugaya A. Kataoka Y. Kanda Y. Taniguchi M.. (2019). OTOF mutation analysis with massively parallel DNA sequencing in 2,265 Japanese sensorineural hearing loss patients. PLoS One 14:e0215932. doi: 10.1371/journal.pone.0215932, PMID: 31095577 PMC6522017

[ref33] Iwasa Y. Nishio S. Y. Yoshimura H. Kanda Y. Kumakawa K. Abe S.. (2013). OTOF mutation screening in Japanese severe to profound recessive hearing loss patients. BMC Med. Genet. 14:95. doi: 10.1186/1471-2350-14-95, PMID: 24053799 PMC3849620

[ref34] Katbamna B. Crumpton T. Patel D. R. (2008). Hearing impairment in children. Pediatr. Clin. N. Am. 55, 1175–1188. doi: 10.1016/j.pcl.2008.07.008 18929059

[ref35] Kelsell D. P. Dunlop J. Stevens H. P. Lench N. J. Liang J. N. Parry G.. (1997). Connexin 26 mutations in hereditary non-syndromic sensorineural deafness. Nature 387, 80–83. doi: 10.1038/387080a0 9139825

[ref36] Kharkovets T. Hardelin J. P. Safieddine S. Schweizer M. El-Amraoui A. Petit C.. (2000). KCNQ4, a K+ channel mutated in a form of dominant deafness, is expressed in the inner ear and the central auditory pathway. Proc. Natl. Acad. Sci. U. S. A. 97, 4333–4338. doi: 10.1073/pnas.97.8.4333, PMID: 10760300 PMC18242

[ref37] Kiang D. T. Jin N. Tu Z. J. Lin H. H. (1997). Upstream genomic sequence of the human connexin26 gene. Gene 199, 165–171. doi: 10.1016/S0378-1119(97)00365-X, PMID: 9358053

[ref38] Kikuchi T. Kimura R. S. Paul D. L. Adams J. C. (1995). Gap junctions in the rat cochlea: immunohistochemical and ultrastructural analysis. Anat Embryol. 191, 101–118. doi: 10.1007/BF00186783, PMID: 7726389

[ref39] Korver A. M. Smith R. J. Van Camp G. Schleiss M. R. Bitner-Glindzicz M. A. Lustig L. R.. (2017). Congenital hearing loss. Nat. Rev. Dis. Primers. 3:16094. doi: 10.1038/nrdp.2016.94, PMID: 28079113 PMC5675031

[ref40] Kujawa S. G. Erostegui C. Fallon M. Crist J. Bobbin R. P. (1994). Effects of adenosine 5′-triphosphate and related agonists on cochlear function. Hear. Res. 76, 87–100. doi: 10.1016/0378-5955(94)90091-4, PMID: 7928720

[ref41] Lammens F. Verhaert N. Devriendt K. Debruyne F. Desloovere C. (2013). Aetiology of congenital hearing loss: a cohort review of 569 subjects. Int. J. Pediatr. Otorhinolaryngol. 77, 1385–1391. doi: 10.1016/j.ijporl.2013.06.002, PMID: 23835162

[ref42] Lee K. Ansar M. Andrade P. B. Khan B. Santos-Cortez R. L. Ahmad W.. (2012). Novel CLDN14 mutations in Pakistani families with autosomal recessive non-syndromic hearing loss. Am. J. Med. Genet. A 158A, 315–321. doi: 10.1002/ajmg.a.34407, PMID: 22246673 PMC3276114

[ref43] Liang C. Zhu Y. Zong L. Lu G. J. Zhao H. B. (2012). Cell degeneration is not a primary causer for Connexin26 (GJB2) deficiency associated hearing loss. Neurosci. Lett. 528, 36–41. doi: 10.1016/j.neulet.2012.08.085, PMID: 22975134 PMC3467974

[ref44] Lieu J. E. C. Kenna M. Anne S. Davidson L. (2020). Hearing loss in children: a review. JAMA 324, 2195–2205. doi: 10.1001/jama.2020.17647, PMID: 33258894

[ref45] Liu X. Han D. Li J. Han B. Ouyang X. Cheng J.. (2010). Loss-of-function mutations in the PRPS1 gene cause a type of nonsyndromic X-linked sensorineural deafness, DFN2. Am. J. Hum. Genet. 86, 65–71. doi: 10.1016/j.ajhg.2009.11.015, PMID: 20021999 PMC2801751

[ref46] Lu Y. C. Wu C. C. Shen W. S. Yang T. H. Yeh T. H. Chen P. J.. (2011). Establishment of a knock-in mouse model with the SLC26A4 c.919-2A>G mutation and characterization of its pathology. PLoS One 6:e22150. doi: 10.1371/journal.pone.0022150, PMID: 21811566 PMC3141011

[ref47] Lynch E. D. Lee M. K. Morrow J. E. Welcsh P. L. Leon P. E. King M. C. (1997). Nonsyndromic deafness DFNA1 associated with mutation of a human homolog of the Drosophila gene diaphanous. Science 278, 1315–1318. doi: 10.1126/science.278.5341.1315, PMID: 9360932

[ref48] Ma J. Ma X. Lin K. Huang R. Bi X. Ming C.. (2023). Genetic screening of a Chinese cohort of children with hearing loss using a next-generation sequencing panel. Hum. Genomics 17:1. doi: 10.1186/s40246-022-00449-1, PMID: 36597107 PMC9811745

[ref49] Macedo-da-Silva J. Marinho C. R. F. Palmisano G. Rosa-Fernandes L. (2020). Lights and shadows of TORCH infection proteomics. Genes (Basel) 11:894. doi: 10.3390/genes11080894, PMID: 32764347 PMC7464470

[ref50] Maljevic S. Wuttke T. V. Seebohm G. Lerche H. (2010). KV7 channelopathies. Pflugers Arch. 460, 277–288. doi: 10.1007/s00424-010-0831-3 20401729

[ref51] Millan J. M. Aller E. Jaijo T. Blanco-Kelly F. Gimenez-Pardo A. Ayuso C. (2011). An update on the genetics of usher syndrome. J. Ophthalmol. 2011:417217. doi: 10.1155/2011/417217 21234346 PMC3017948

[ref52] Miyajima H. Moteki H. Day T. Nishio S. Y. Murata T. Ikezono T.. (2020). Novel ACTG1 mutations in patients identified by massively parallel DNA sequencing cause progressive hearing loss. Sci. Rep. 10:7056. doi: 10.1038/s41598-020-63690-5, PMID: 32341388 PMC7184572

[ref53] Morin M. Bryan K. E. Mayo-Merino F. Goodyear R. Mencia A. Modamio-Hoybjor S.. (2009). In vivo and in vitro effects of two novel gamma-actin (ACTG1) mutations that cause DFNA20/26 hearing impairment. Hum. Mol. Genet. 18, 3075–3089. doi: 10.1093/hmg/ddp249, PMID: 19477959 PMC2714729

[ref54] Morton C. C. Nance W. E. (2006). Newborn hearing screening--a silent revolution. N. Engl. J. Med. 354, 2151–2164. doi: 10.1056/NEJMra050700, PMID: 16707752

[ref55] Munoz D. J. Thorne P. R. Housley G. D. Billett T. E. Battersby J. M. (1995). Extracellular adenosine 5′-triphosphate (ATP) in the endolymphatic compartment influences cochlear function. Hear. Res. 90, 106–118. doi: 10.1016/0378-5955(95)00152-3, PMID: 8974987

[ref56] Nance W. E. (2003). The genetics of deafness. Ment. Retard. Dev. Disabil. Res. Rev. 9, 109–119. doi: 10.1002/mrdd.10067 12784229

[ref57] Neuhaus C. Lang-Roth R. Zimmermann U. Heller R. Eisenberger T. Weikert M.. (2017). Extension of the clinical and molecular phenotype of DIAPH1-associated autosomal dominant hearing loss (DFNA1). Clin. Genet. 91, 892–901. doi: 10.1111/cge.12915, PMID: 27808407

[ref58] Nie L. (2008). KCNQ4 mutations associated with nonsyndromic progressive sensorineural hearing loss. Curr. Opin. Otolaryngol. Head Neck Surg. 16, 441–444. doi: 10.1097/MOO.0b013e32830f4aa3, PMID: 18797286 PMC2743278

[ref59] Pangrsic T. Lasarow L. Reuter K. Takago H. Schwander M. Riedel D.. (2010). Hearing requires otoferlin-dependent efficient replenishment of synaptic vesicles in hair cells. Nat. Neurosci. 13, 869–876. doi: 10.1038/nn.2578, PMID: 20562868

[ref60] Peters L. M. Anderson D. W. Griffith A. J. Grundfast K. M. San Agustin T. B. Madeo A. C.. (2002). Mutation of a transcription factor, TFCP2L3, causes progressive autosomal dominant hearing loss, DFNA28. Hum. Mol. Genet. 11, 2877–2885. doi: 10.1093/hmg/11.23.2877, PMID: 12393799

[ref61] Petrof G. Nanda A. Howden J. Takeichi T. McMillan J. R. Aristodemou S.. (2014). Mutations in GRHL2 result in an autosomal-recessive ectodermal dysplasia syndrome. Am. J. Hum. Genet. 95, 308–314. doi: 10.1016/j.ajhg.2014.08.001, PMID: 25152456 PMC4157147

[ref62] Pollock L. M. McDermott B. M. Jr. (2015). The cuticular plate: a riddle, wrapped in a mystery, inside a hair cell. Birth Defects Res. C Embryo Today 105, 126–139. doi: 10.1002/bdrc.21098, PMID: 26104653

[ref63] Rabionet R. Zelante L. Lopez-Bigas N. D'Agruma L. Melchionda S. Restagno G.. (2000). Molecular basis of childhood deafness resulting from mutations in the GJB2 (connexin 26) gene. Hum. Genet. 106, 40–44. PMID: 10982180 10.1007/s004390051007

[ref64] Reardon W. Lewis N. Hughes H. E. (1993). Consanguinity, cardiac arrest, hearing impairment, and ECG abnormalities: counselling pitfalls in the Romano-Ward syndrome. J. Med. Genet. 30, 325–327. doi: 10.1136/jmg.30.4.325, PMID: 8487283 PMC1016346

[ref65] Rhodes C. R. Hertzano R. Fuchs H. Bell R. E. de Angelis M. H. Steel K. P.. (2004). A Myo7a mutation cosegregates with stereocilia defects and low-frequency hearing impairment. Mamm. Genome 15, 686–697. doi: 10.1007/s00335-004-2344-x, PMID: 15389316

[ref66] Ritter K. E. Martin D. M. (2019). Neural crest contributions to the ear: implications for congenital hearing disorders. Hear. Res. 376, 22–32. doi: 10.1016/j.heares.2018.11.005, PMID: 30455064 PMC6456423

[ref67] Rivas A. Francis H. W. (2005). Inner ear abnormalities in a Kcnq1 (Kvlqt1) knockout mouse: a model of Jervell and Lange-Nielsen syndrome. Otol. Neurotol. 26, 415–424. doi: 10.1097/01.mao.0000169764.00798.84, PMID: 15891643

[ref68] Robbins J. (2001). KCNQ potassium channels: physiology, pathophysiology, and pharmacology. Pharmacol. Ther. 90, 1–19. doi: 10.1016/S0163-7258(01)00116-4 11448722

[ref69] Schraders M. Haas S. A. Weegerink N. J. Oostrik J. Hu H. Hoefsloot L. H.. (2011). Next-generation sequencing identifies mutations of SMPX, which encodes the small muscle protein, X-linked, as a cause of progressive hearing impairment. Am. J. Hum. Genet. 88, 628–634. doi: 10.1016/j.ajhg.2011.04.012, PMID: 21549342 PMC3146715

[ref70] Smith R. J. (2004). Clinical application of genetic testing for deafness. Am. J. Med. Genet. A 130A, 8–12. doi: 10.1002/ajmg.a.30053 15368487

[ref71] Smith R. J. Bale J. F. Jr. White K. R. (2005). Sensorineural hearing loss in children. Lancet 365, 879–890. doi: 10.1016/S0140-6736(05)71047-3 15752533

[ref72] Soleimani M. Greeley T. Petrovic S. Wang Z. Amlal H. Kopp P.. (2001). Pendrin: an apical cl-/OH-/HCO3- exchanger in the kidney cortex. Am. J. Physiol. Renal Physiol. 280, F356–F364. doi: 10.1152/ajprenal.2001.280.2.F356, PMID: 11208611

[ref73] Sundstrom R. A. Van Laer L. Van Camp G. Smith R. J. (1999). Autosomal recessive nonsyndromic hearing loss. Am. J. Med. Genet. 89, 123–129. doi: 10.1002/(SICI)1096-8628(19990924)89:3<123::AID-AJMG2>3.0.CO;2-P 10704186

[ref74] Teubner B. Michel V. Pesch J. Lautermann J. Cohen-Salmon M. Sohl G.. (2003). Connexin30 (Gjb6)-deficiency causes severe hearing impairment and lack of endocochlear potential. Hum. Mol. Genet. 12, 13–21. doi: 10.1093/hmg/ddg001, PMID: 12490528

[ref75] van Beeck Calkoen E. A. Engel M. S. D. van de Kamp J. M. Yntema H. G. Goverts S. T. Mulder M. F.. (2019). The etiological evaluation of sensorineural hearing loss in children. Eur. J. Pediatr. 178, 1195–1205. doi: 10.1007/s00431-019-03379-8, PMID: 31152317 PMC6647487

[ref76] Van Laer L. McGuirt W. T. Yang T. Smith R. J. Van Camp G. (1999). Autosomal dominant nonsyndromic hearing impairment. Am. J. Med. Genet. 89, 167–174. doi: 10.1002/(SICI)1096-8628(19990924)89:3<167::AID-AJMG7>3.0.CO;2-V 10704191

[ref77] van Wijk E. Krieger E. Kemperman M. H. De Leenheer E. M. Huygen P. L. Cremers C. W.. (2003). A mutation in the gamma actin 1 (ACTG1) gene causes autosomal dominant hearing loss (DFNA20/26). J. Med. Genet. 40, 879–884. doi: 10.1136/jmg.40.12.879, PMID: 14684684 PMC1735337

[ref78] Wang Y. Jin Y. Zhang Q. Xiong Y. Gu X. Zeng S.. (2023). Research progress in delineating the pathological mechanisms of GJB2-related hearing loss. Front. Cell. Neurosci. 17:1208406. doi: 10.3389/fncel.2023.1208406, PMID: 37333892 PMC10272732

[ref79] Wang J. J. Li Y. (2016). KCNQ potassium channels in sensory system and neural circuits. Acta Pharmacol. Sin. 37, 25–33. doi: 10.1038/aps.2015.131, PMID: 26687932 PMC4722976

[ref80] Wen Z. Zhu H. Li Z. Zhang S. Zhang A. Zhang T.. (2019). A knock-in mouse model of Pendred syndrome with Slc26a4 L236P mutation. Biochem. Biophys. Res. Commun. 515, 359–365. doi: 10.1016/j.bbrc.2019.05.157, PMID: 31155292

[ref81] Yasunaga S. Grati M. Chardenoux S. Smith T. N. Friedman T. B. Lalwani A. K.. (2000). OTOF encodes multiple long and short isoforms: genetic evidence that the long ones underlie recessive deafness DFNB9. Am. J. Hum. Genet. 67, 591–600. doi: 10.1086/303049, PMID: 10903124 PMC1287519

[ref82] Yasunaga S. Grati M. Cohen-Salmon M. El-Amraoui A. Mustapha M. Salem N.. (1999). A mutation in OTOF, encoding otoferlin, a FER-1-like protein, causes DFNB9, a nonsyndromic form of deafness. Nat. Genet. 21, 363–369. doi: 10.1038/7693, PMID: 10192385

[ref83] Zong L. Guan J. Ealy M. Zhang Q. Wang D. Wang H.. (2015). Mutations in apoptosis-inducing factor cause X-linked recessive auditory neuropathy spectrum disorder. J. Med. Genet. 52, 523–531. doi: 10.1136/jmedgenet-2014-102961, PMID: 25986071 PMC4518735

[ref84] Zong Y. J. Liu X. Z. Tu L. Sun Y. (2023). Cytomembrane trafficking pathways of Connexin 26, 30, and 43. Int. J. Mol. Sci. 24:10349. doi: 10.3390/ijms241210349, PMID: 37373495 PMC10298996

